# Supplementary material for: Online Left-Hemispheric In-Phase Frontoparietal Theta tACS for the Treatment of Negative Symptoms of Schizophrenia
Source: J Pers Med. 2021 Oct 29;11(11):1114. doi: 10.3390/jpm11111114 (PMC8625275; doi:10.3390/jpm11111114)
Supplement: Supplementary file 1 [file jpm-11-01114-s001.zip › jpm-1416476-supplementary.pdf]

Supplementary Materials

This supplementary information contains: Supplementary Text/Tables, Supplementary Figures, and References.

1. Supplementary Text

Randomization and Blinding

A study coordinator not involved in the execution of the trial carried out the randomization by using an online randomization tool for clinical trials (https://www.sealedenvelope.com/). Participants were assigned to the active or sham stimulation in a 1:1 ratio using blocked randomization with randomly permuted blocks of four. The allocation concealment system was performed through central randomization, in which the researcher contacted the study coordinator after enrolling and registering the participant. The allocation concealment was further ensured by the administration of tACS using “study mode of the device” in which a five-digit numerical code specific to individual participant was entered into the devices (Eldith DC stimulator Plus, NeuroConn, Ilmenau, Germany) that resulted in either active or sham stimulation [1]; i.e., the researcher got the randomization code and a unique five-digit numerical code for each participant from the study coordinator while the tACS administrators entered the code for study mode into the devices. The study coordinator had continuous access to the randomization list and unblinded the trial after the final visit of the last participant. Not until the trial was unblinded did the participants, tACS administrators, researchers and clinical raters know the actual stimulation types. The only one reason for premature code-breaking was that any suspected unexpected serious adverse reaction (SUSAR) occurred. The study coordinator would disclose the treatment code before a SUSAR was reported to the local institutional review boards (IRB) and the health agency. Once the masking code was broken, the treatment for the participant would be discontinued.

Effectiveness of blinding

Each participant was asked to answer the question of whether he or she had received active tACS or sham stimulation shortly after the 1st session of stimulation and at the end of treatment. After the trial was unblinded, analyses showed that 83.3% of participants receiving active tACS and 72.2% of those receiving sham guessed they had received active stimulation shortly after the 1st session of stimulation. Fisher's exact test revealed no statistically significant between-group differences ($p = 0.69$), suggesting satisfactory effectiveness of our blinding protocol. Similarly, active guesses between the two groups were not different at the end of treatment ($p = 0.53$)

The definition of dropout

Dropout was considered after the absence in two consecutive tACS sessions or declined consent to participate after receiving the first tACS session.

Dual n-back task

Dual n-back task [2] in Brain Workshop software v4.8.4 was used for computerized WM training. In this task, squares at 8 different locations showed up sequentially (stimulus length, 500 ms; inter-stimulus interval, 2,500 ms) on a computer screen every 3 seconds. Simultaneously with the presentation of the squares, one of eight consonants showed up sequentially through a speaker.

Participants had to judge whether the location of a square and the consonant they heard matched the one n-back before (the same n value for both visual and auditory targets). Each training session had 20 blocks (each block consisting of 20 + n trials) while each block included six auditory and six visual targets (four appearing in only one modality, and two appearing in both modalities simultaneously) whose locations were random. Participants had to make responses manually by pressing the mouse left-click button for visual targets and the right-click button for auditory targets. No responses were required for non-targets. If a target was correctly detected, a flashing green light signal would show up as positive feedback. If a target was falsely detected, a flashing blue light signal would show up as negative feedback. The dual n-back training was designed to continuously vary its difficulty by modifying the WM load (i.e., the level of n) and thereby track the participants' performance. Each training session began at n = 1. Participants' performance was analyzed after each block and the level of n for the next block would be adapted according to the following principle. The level of n increased by 1 in the next block if the mistakes per modality made by the participant were < 3. Conversely, n decreased by 1 if the mistakes per modality made by the participant were > 5. In all other cases, the n was kept unchanged. Participants came to the laboratory and took part in the WM training sessions twice daily for 5 weekdays (total 10 sessions), with each session lasting for approximately 25 minutes. The time interval between twice-daily sessions was >3 hours. It was known that 10-session dual n-back training alone failed to significantly improve severity of negative symptoms in schizophrenia (Li et al., 2019a).

The assessment of psychopathological symptoms

The Chinese version of the Positive and Negative Syndrome Scale (PANSS) was administered to assess the severity of psychopathology. A single rater (HAC) scored each item by deciding which anchoring point in the 7-point scale (1 = absent; 7 = extreme) best characterized the presentation of the psychopathological symptoms. The 30-item scale contains three subscales (positive, negative and general psychopathology) [3] and PANSS factor scores for positive symptoms (PANSS-FSPS, the sum of items P1, P3, P5, P6, and G9), negative symptoms (PANSS-FSNS, the sum of items of N1-N4, N6, G7, and G16), cognitive component (the sum of items P2, N5, and G11), excited component (the sum of items P4, P7, G8, and G14), and emotional/depressed component (the sum of items G2, G3, G4, and G6) [4, 5]. Two subdomains of negative symptoms were identified from the PANSS: Expressive Negative Symptoms (Exp Neg, the sum of items of N1, N3, N6, G5, G7 and G13) and social amotivation (Soc Amot, the sum of items of N2, N4, and G16) [6]. Also, negative symptoms were assessed by the same rater using the Scale for the Assessment of Negative Symptoms (SANS), a 25 item, 6-point scale that rates the five domains of affective blunting, alogia, avolition/apathy, anhedonia/asociality, and attention from absent (a score of 0) to severe (a score of 5) [7].

The assessment of insight levels

The abbreviated version of the Scale to Assess Unawareness in Mental Disorder in schizophrenia (SUMD) is an interviewer-rating scale [8] to assess patients' current states of awareness, containing 9 items which are divided into 3 dimensions of awareness of the disease (item 1-3: a mental disorder, consequences of a mental disorder and effects of drugs), awareness of positive symptoms

(item 4-6: hallucinatory experiences, delusional ideas, and disorganized thoughts) and awareness of negative symptoms (item 7-9: blunted affect, anhedonia, and lack of sociability). Score 0 (not applicable), 1 (aware), 2 (somewhat aware/unaware) or 3 (severely unaware) is rated on each item. The average score on each dimension is calculated and then linearized on a 0–100 scale (from the lowest to the highest level of unawareness). The Taiwanese version of the Self-Appraisal of Illness Questionnaire (SAIQ) [9], translated from the original SAIQ [10], was used to assess subjective experiences and attitudes toward mental illness and experience of psychiatric treatment. It comprises 17 items on which the patients rated from a score of 1 (do not agree at all) to 4 (completely agree). The translated SAIQ contains three subscales (the presence/outcome, need for treatment, and worry). All subscales scores were summed to yield a total score of SAIQ, with a higher score indicating more awareness of the mental illness. The Taiwanese version of the Beck Cognitive Insight Scale (BCIS) [11, 12], translated from the original BCIS [13], was used to assess patients' cognitive insight. The scale contains 15 items, which are divided into 2 subscales of reflective attitude (9 items) and certain attitude (6 items). The reflective attitude subscale score minus the certain attitude subscale score yields R-C index, which indicates the level of cognitive insight. Higher R-C index scores represent better cognitive insight.

The assessment of psychosocial functioning

The Taiwanese version of the Personal and Social Performance scale (PSP) [14], translated from the original PSP scale [15], was used by a rater (HAC) to assess the following domains of psychosocial functioning: socially useful activities, personal and social relationships, self-care, and disturbing and aggressive behaviors. The summary instruction table of PSP scale defines the final global score, ranging from 1 to 100, with a higher score indicating better psychosocial function. The self-reported version of the graphic personal and social performance scale (SRG-PSP) was used to assess psychosocial function outcomes [16]. It comprises 22 items with each item rated from 1 (seldom) to 3 (always) and is divided into four domains, in which lower domain scores represent poorer functioning in socially useful activities (a), personal and social relationships (b), and self-care (c), but less disturbing and aggressive behaviors (d). The sum of domain (a, b, c) scores minus domain (d) score yields the global score of the SRG-PSP. A lower global score indicates poorer psychosocial functioning.

The assessment of perceived quality of life

Subjective quality of life (QoL) was assessed using the Chinese version of the Schizophrenia Quality of Life Scale Revision Four (SQLS-R4), a valid and reliable self-administered questionnaire comprising 33 items in two domains (psychosocial and vitality) [17]. All items are coded on a scale of 0–4 according to the frequency of occurrence during the previous 7 days (0 = always, 4 = never) with 4 exceptional items that are reverse-coded (0 = never, 4 = always). A higher score implies better health-related QoL.

Dual 2-back task

The dual 2-back task is an optional mode of the dual n-back task [2] from Brain Workshop software v4.8.4, in which a fixed n-level of 2 is used. During the task, squares at 8 different locations showed up sequentially (stimulus length, 500 ms; inter-stimulus interval, 2500 ms) on a computer screen

every 3 seconds. Simultaneously with the presentation of the squares, one of eight consonants showed up sequentially through a speaker. Participants had to judge whether the location of a square and the consonant they heard matched the one 2-back before (the same for both visual and auditory targets). The task was presented 5 times in a block design with 21 trials (63s) in each block (Totally 5 blocks, 315 s). Each block included six auditory and six visual targets (four appearing in only one modality, and two appearing in both modalities simultaneously) representing stimuli that matched the stimuli 2 positions back in the sequence. Participants had to make responses manually by pressing the mouse left-click button for visual targets and the right-click button for auditory targets. No responses were required for non-targets. If a target was correctly detected, a flashing green light signal would show up as positive feedback. If a target was falsely detected, a flashing blue light signal would show up as negative feedback. Accuracy was calculated as the proportion of correct responses in each block and the data of accuracy from all five blocks were averaged.

2. Supplementary Tables

Table S1. Other baseline clinical characteristics of the sample (complementary material).

Characteristics	Active tACS (N=18)	Sham (N=18)	t/U or χ2/Fisher's	p-value
PSP				
Social useful activities	3.33±0.59	3.56±0.62	128.00	0.23
Personal and social relationships	3.17±0.79	3.67±0.59	108.50	0.06
Self-care	1.89±0.58	2.06±0.64	140.00	0.42
Disturbing and aggressive behavior	1.78±0.65	2.17±0.79	116.00	0.12
Global score	56.06±9.66	49.72±9.63	90.00	0.020
SRG-PSP				
Social useful activities	10.44±3.05	9.89±3.34	146.00	0.61
Personal and social relationships	14.44±2.96	12.72±2.37	-1.93	0.06
Self-care	16.67±1.71	16.16±2.64	154.50	0.80
Disturbing and aggressive behavior	5.56±0.86	6.22±2.26	157.50	0.87
Global score	36.00±6.46	32.56±6.57	-1.59	0.12
SUMD				
Awareness of disease	63.58±19.36	72.22±21.64	1.26	0.22
Awareness of positive symptoms	39.51±13.86	47.53±18.20	1.49	0.15
Awareness of negative symptoms	69.14±15.51	77.78±17.46	118.50	0.15
SAIQ				
Total score	50.33±7.12	53.72±7.17	1.42	0.16
Worry	19.78±4.39	21.83±4.57	1.38	0.18
Need treatment	16.00±1.94	17.61±2.38	92.00	0.025
Presence/outcome	14.56±2.50	14.28±3.01	151.50	0.74
BCIS				
BCIS-R	24.11±4.36	23.50±5.60	149.00	0.68

BCIS-C	16.61±2.55	15.72±3.41	172.50	0.74
R-C index	7.50±4.85	7.78±6.28	151.50	0.74
SQLS-R4				
Total score	62.67±20.70	57.56±24.45	-0.68	0.50
Psychosocial domain	37.50±15.40	33.28±17.18	-0.78	0.44
Vitality domain	25.17±6.94	24.28±8.57	-0.34	0.74

Abbreviations: tACS, Online theta transcranial alternating current stimulation; PSP, Personal and Social Performance scale; SRG-PSP, Self-reported version of the graphic Personal and Social Performance scale; SUMD, the abbreviated version of the Scale to Assess Unawareness in Mental Disorder; SAIQ, The Taiwanese version of Self-Appraisal of Illness Questionnaire; BCIS, The Taiwanese version of Beck's Cognitive Insight Scale; BCIS-R, Self-reflectiveness subscale of BCIS; BCIS-C, Self-certainty subscale of BCIS; SQLS-R4, the Schizophrenia Quality of Life Scale Revision Four.

Notes: Data are presented as means ± standard deviations unless otherwise stated;
Significant p-values are presented in bold.

Table S2. Neurocognitive assessments at baseline

Characteristics	Active tACS (N=18)	Sham (N=18)	t/U or χ2/Fisher's	p-value
Dual 2-back task accuracy, %	19.72±9.36	19.39±11.57	0.10	0.93
WCST				
Trials Completed	98.00±23.98	102.65±25.30	142.00	0.71
Trials Correct	75.28±10.34	70.35±17.93	-1.00	0.32
Total Errors	22.72±15.87	32.29±27.73	124.00	0.34
Perseverative Responses	0.21±0.11	0.28±0.20	125.00	0.36
Perseverative Errors	12.61±8.98	24.47±27.49	126.50	0.37
Nonperseverative Errors	11.22±7.51	20.59±21.76	127.50	0.39
Conceptual Level Responses	11.50±9.02	11.71±9.63	148.00	0.87
Categories Completed	68.94±6.88	61.82±23.32	123.50	0.33
CPT II				
Omission errors	11.67±20.39	19.67±39.24	130.50	0.32
Commission errors	12.00±9.06	13.50±7.96	0.53	0.60
Hit RT (ms)	467.36±81.07	490.48±126.16	155.00	0.83
Hit RT SE	8.83±5.77	10.80±10.14	136.00	0.41
Variability (Variability of SE)	15.28±14.21	18.35±21.81	146.00	0.61
Detectability (d')	0.99±0.62	0.73±0.55	117.00	0.15
Response Style (ß)	1.11±1.01	1.62±1.66	132.00	0.34
Perseverations	2.67±5.29	4.17±7.90	144.50	0.57
Hit RT Block Change	0.02±0.03	0.01±0.03	-1.02	0.32
Hit SE Block Change	0.04±0.10	0.04±0.10	-0.07	0.95

Hit RT ISI Change	0.06±0.04	0.08±0.04	113.50	0.12
Hit SE ISI Change	0.05±0.13	0.07±0.14	0.42	0.68
Digit span				
Forward	12.89±3.29	12.22±3.49	142.50	0.53
Backward	6.78±2.88	6.22±4.66	121.00	0.19
FTT				
Dominant finger	56.38±10.32	54.42±10.41	-0.57	0.57
Non-dominant finger	50.18±6.90	50.67±9.33	156.50	0.86
TOL				
TOL accuracy	3.78±2.32	3.33±2.38	147.00	0.63
TOL time	248.94±95.48	223.11±87.56	142.00	0.53
TOL score	0.72±0.89	0.94±1.66	161.00	0.97
CTT				
Color 1 (time in seconds)	56.53±27.26	51.75±24.29	-0.55	0.58
Color 2 (time in seconds)	106.39±42.31	103.54±37.35	161.00	0.98
Stroop Interference Test				
Stroop Interference tendency				
Reading interference tendency	0.34±0.34	0.26±0.31	136.00	0.41
Naming interference tendency	0.36±0.32	0.30±0.31	141.50	0.52

Abbreviations: tACS, Online theta transcranial alternating current stimulation.; WCST, Wisconsin Card Sorting Test; CPT-II; RT, reaction time; SE, standard error; ISI, inter-stimulus interval; FTT, Finger Tapping Test; TOL, Tower of London test; CTT, Color Trails Test.
Notes: Data are presented as means ± standard deviations unless otherwise stated; Neurocognitive function was assessed using standardized tests administered by a well-trained examiner and participants were instructed to abstain from caffeine for at least 24 h before the assessments.

Wisconsin Card Sorting Test (WCST). The test was used to measure executive functions including abstract reasoning ability, cognitive flexibility in response to changing environmental contingencies [18].

Connors' Continuous Performance Test –2nd Edition (CPT-II). The examinee performed the test to assess participants' concentration, sustained attention, response inhibition and impulsivity [19].

Digit span, the digit span forward and backward subtests of the Wechsler Memory Scale-III. The examinee performed the test to assess participants' load capacity of working memory [20].

Finger Tapping Test (FTT). The test was used to measure psychomotor speed and the level of motor coordination[21].

Tower of London test (TOL). The Tower of London-Drexel University Test 2nd Edition (TOLDXtm)

was administered to assess executive functioning, especially regarding the ability of planning, processing, and problem-solving skills [22]. TOL accuracy indicates the total correct score in the TOL. TOL time indicates the total time spent in the TOL. TOL score indicates the total rule violations in the TOL.

Color Trails Test (CTT). The test was used to assess attention and visuomotor processing speed, attentional set-shifting, executive function and visuospatial working memory [23].

Stroop Interference Test. The computerized test on the Vienna Test system was used to assess selective attention and cognitive flexibility [24].

Table S3. Comparisons of the changes over time in psychopathology score between participants treated with active tACS and those with sham stimulation.

Items	Time effect	Group effect	Interaction effect
PANSS negative symptoms subscale score	$F_{3,32}$=11.46, p<0.001	$F_{1,34}$=2.67, p=0.11	$F_{3,32}$=3.62, p=0.023
PANSS total score	$F_{3,32}$=9.85, p<0.001	$F_{1,34}$=2.91, p=0.10	$F_{3,32}$=5.19, p=0.005
PANSS five-factor score			
PANSS-FSPS	$F_{3,32}$=1.40, p=0.26	$F_{1,34}$=0.18, p=0.67	$F_{3,32}$=0.96, p=0.42
PANSS-FSNS	$F_{3,32}$=8.22, p<0.001	$F_{1,34}$=1.52, p=0.23	$F_{3,32}$=2.98, p=0.046
Excited	$F_{3,32}$=2.15, p=0.11	$F_{1,34}$=0.10, p=0.75	$F_{3,32}$=1.88, p=0.15
Cognitive	$F_{3,32}$=23.72, p<0.001	$F_{1,34}$=8.20, p=0.007	$F_{3,32}$=14.37, p<0.001
Emotional/depressed	$F_{3,32}$=0.74, p=0.54	$F_{1,34}$=0.00, p=0.98	$F_{3,32}$=1.70, p=0.19
PANSS two-subdomain score of negative symptoms			
Exp Neg	$F_{3,32}$=5.95, p=0.002	$F_{1,34}$=0.36, p=0.55	$F_{3,32}$=0.33, p=0.81
Soc Amot	$F_{3,32}$=5.93, p=0.002	$F_{1,34}$=3.56, p=0.07	$F_{3,32}$=3.14, p=0.039
SANS	$F_{3,32}$=10.22, p<0.001	$F_{1,34}$=1.64, p=0.21	$F_{3,32}$=3.10, p=0.04

Abbreviations: tACS, Online theta transcranial alternating current stimulation.; PANSS, Positive and Negative Syndrome Scale; FSNS, Factor Score for Negative Symptoms; FSPS, Factor Score for Positive Symptoms; Exp Neg, Expressive Negative symptoms; Soc Amot, Social Amotivation; SANS, Scale for the Assessment of Negative Symptoms

Notes: Significant P-values for interaction effects are presented in bold.

Table S4. Associations between the daily doses of concomitant medications and improvement in negative symptoms.

	Changes in PANSS_N scores (baseline to after tACS)		Changes in PANSS_N scores (baseline to one-week F/U)		Changes in PANSS_N scores (baseline to one-month F/U)	
Daily doses of concomitant medications	r	p	r	p	r	p
Daily dose of antipsychotic medicationsa	-0.08	0.76	0.28	0.26	0.40	0.10
Daily dose of anticholinergic antiparkinsonian medicationsb	-0.16	0.53	0.04	0.88	0.14	0.59
Daily dose of sedative-hypnoticsc	0.61	0.007	0.56	0.016	0.44	0.07

Abbreviations: PANSS_N, negative symptoms subscale of the Positive and Negative Syndrome Scale; tACS, online theta transcranial alternating current stimulation.;

aThe daily dose of antipsychotic medications was converted to olanzapine equivalent

bThe daily dose of anticholinergic antiparkinsonian medications was converted to biperiden equivalent

cThe daily dose of sedative-hypnotics was converted to diazepam equivalent

P values that reach the corrected significance level (false discovery rate method) are in bold.

Table S5. Treatment-emergent adverse events experienced by participants in active tACS group and sham group.

Treatment-emergent adverse events	Grouping	
	Active tACS (n=18)	Sham (n=18)
Tingling	9 (50.00%)	4 (22.20%)
Sleepiness	8 (44.40%)	10 (55.60%)
Scalp pain	6 (33.30%)	4 (22.20%)
Dizziness	3 (16.70%)	2 (11.10%)
Trouble concentrating	3 (16.70%)	3 (16.70%)
Burning sensation	2 (11.10%)	0 (0.00%)
Itching	1 (5.60%)	1 (5.60%)
Headache	1 (5.60%)	0 (0.00%)
Head pressure	0 (0.00%)	1 (5.60%)
Neck pain	0 (0%)	0 (0%)
Skin redness	0 (0%)	0 (0%)
Acute mood change	0 (0%)	0 (0%)

Abbreviations: tACS, online theta transcranial alternating current stimulation.

Notes: Each number represents the number (and percent) of participants who reported any given side effect at least once after the first session of stimulation. Mean total side effect scores over the 10 sessions of tACS (calculated using a score of 1 for each side effect reported by a participant in each session of tACS) was 0.39±0.25 in the active tACS group and 0.28±0.23 in the sham group. There was no significant difference in mean total side effect score between active vs. sham group (Student's t-test, t=1.34, P=0.19).

Table S6. Comparisons of the changes over time in other secondary outcomes between participants treated with active tACS and those with sham stimulation.

Variables	Time effect	Group effect	Interaction effect

Researcher-rated measures at four different time points (at baseline, shortly after 10-session stimulation, and at one-week and one-month follow-ups)			
PSP			
Social useful activities	F3,32=8.52, p<0.001	F1,34=6.09, p=0.019	F3,32=4.46, p=0.01
Personal and social relationships	F3,32=0.63, p=0.60	F1,34=10.09, p=0.003	F3,32=2.94, p=0.048
Self-care	F3,32=0.89, p=0.46	F1,34=3.54, p=0.07	F3,32=1.89, p=0.15
Disturbing and aggressive behavior	F3,32=3.18, p=0.037	F1,34=6.16, p=0.018	F3,32=1.31, p=0.29
Global score	F3,32=6.37, p=0.002	F1,34=9.15, p=0.005	F3,32=6.31, p=0.002
SUMD			
Awareness of disease	F2,33=1.18, p=0.32	F1,34=2.26, p=0.14	F2,33=1.80, p=0.18
Awareness of positive symptoms	F3,32=0.48, p=0.70	F1,34=2.63, p=0.11	F3,32=1.62, p=0.21
Awareness of negative symptoms	F2,33=1.37, p=0.27	F1,34=3.53, p=0.07	F2,33=0.63, p=0.54
Self-report measures at three different time points (at baseline, shortly after 10-session stimulation, and at one-week follow-up)			
SRG-PSP			
Social useful activities	F2,33=1.77, p=0.19	F1,34=1.15, p=0.29	F2,33=6.31, p=0.005
Personal and social relationships	F2,33=1.62, p=0.21	F1,34=1.27, p=0.27	F2,33=1.83, p=0.18
Self-care	F2,33=1.00, p=0.38	F1,34=0.38, p=0.54	F2,33=0.05, p=0.95
Disturbing and aggressive behavior	F2,33=0.31, p=0.73	F1,34=3.38, p=0.08	F2,33=0.90, p=0.42
Global score	F2,33=3.03, p=0.06	F1,34=2.14, p=0.15	F2,33=1.44, p=0.25
SAIQ			
Total score	F2,33=1.54, p=0.23	F1,34=0.34, p=0.56	F2,33=1.42, p=0.26
Worry	F2,33=1.29, p=0.29	F1,34=0.21, p=0.65	F2,33=0.85, p=0.44
Need treatment	F2,33=1.49, p=0.24	F1,34=3.33, p=0.08	F2,33=2.63, p=0.09
Presence/outcome	F2,33=0.48, p=0.63	F1,34=0.48, p=0.49	F2,33=0.18, p=0.84
BCIS			
BCIS-R	F2,33=0.15, p=0.86	F1,34=0.66, p=0.42	F2,33=1.88, p=0.17

BCIS-C	F2,33=0.37, p=0.69	F1,34=0.23, p=0.64	F2,33=4.72, p=0.016
R-C index	F2,33=0.18, p=0.84	F1,34=0.15, p=0.70	F2,33=0.13, p=0.88
SQLS			
Total	F2,33=1.67, p=0.20	F1,34=0.43, p=0.52	F2,33=2.46, p=0.10
Psychosocial	F2,33=0.31, p=0.28	F1,34=0.58, p=0.45	F2,33=2.54, p=0.10
Vitality	F2,33=1.61, p=0.20	F1,34=0.15, p=0.70	F2,33=0.52, p=0.60

Abbreviations: tACS, online theta transcranial alternating current stimulation; PSP, Personal and Social Performance scale; SRG-PSP, Self-Reported version of the Graphic Personal and Social Performance scale; SUMD, the abbreviated version of the Scale to Assess Unawareness in Mental Disorder; SAIQ, the Taiwanese version of Self-Appraisal of Illness Questionnaire; BCIS, the Taiwanese version of Beck's Cognitive Insight Scale; BCIS-R, Self-reflectiveness subscale of BCIS; BCIS-C, Self-certainty subscale of BCIS; SQLS-R4, the Schizophrenia Quality of Life Scale Revision Four.

Notes: Significant P-values for interaction effects are presented in bold.

Table S7. Comparisons of the changes over time in a variety of neurocognitive performance between participants treated with active tACS and those with sham stimulation.

Items	Time effect	Group effect	Interaction effect
Measures at four different time points (at baseline, shortly after 10-session stimulation, and at one-week and one-month follow-ups)			
Dual 2-back task accuracy, %	F3,32=30.72, p<0.001	F1,34=6.89, p=0.013	F3,32=7.27, p=0.001
Measures at three different time points (at baseline, shortly after 10-session stimulation, and at one-week follow-up)			
WCST			
Trials Completed	F2,32=1.18, p=0.32	F1,33=0.87, p=0.36	F2,32=0.03, p=0.97
Trials Correct	F2,32=0.54, p=0.59	F1,33=0.05, p=0.83	F2,32=2.27, p=0.12
Total Errors	F2,32=0.30, p=0.75	F1,33=1.38, p=0.25	F2,32=1.10, p=0.34
Perseverative Responses	F2,32=1.09, p=0.35	F1,33=2.21, p=0.15	F2,32=1.36, p=0.27
Perseverative Errors	F2,32=1.09, p=0.35	F1,33=2.13, p=0.15	F2,32=1.39, p=0.27
Nonperseverative Errors	F2,32=0.22, p=0.81	F1,33=0.26, p=0.61	F2,32=0.15, p=0.87
Conceptual Level Responses	F2,32=0.72, p=0.49	F1,33=1.93, p=0.17	F2,32=1.72, p=0.20
Categories Completed	F2,32=0.21, p=0.81	F1,33=2.10, p=0.16	F2,32=0.33, p=0.72
CPT II			
Omission errors	F2,32=2.99, p=0.07	F1,33=1.28, p=0.27	F2,32=0.35, p=0.71
Commission errors	F2,32=0.81, p=0.45	F1,33=0.19, p=0.67	F2,32=1.66, p=0.21
Hit RT (ms)	F2,32=1.96, p=0.16	F1,33=0.54, p=0.47	F2,32=0.36, p=0.70
Hit RT SE	F2,32=2.47, p=0.10	F1,33=1.34, p=0.26	F2,32=0.42, p=0.66
Variability (Variability of SE)	F2,32=1.20, p=0.31	F1,33=1.18, p=0.29	F2,32=0.51, p=0.61
Detectability (d')	F2,32=0.85, p=0.44	F1,33=1.12, p=0.30	F2,32=1.86, p=0.17
Response Style (ß)	F2,32=0.19, p=0.83	F1,33=1.09, p=0.30	F2,32=0.95, p=0.40
Perseverations	F2,32=1.74, p=0.19	F1,33=1.24, p=0.27	F2,32=0.69, p=0.51

Hit RT Block Change	F2,32=2.52, p=0.10	F1,33=0.02, p=0.90	F2,32=1.68, p=0.20
Hit SE Block Change	F2,32=2.96, p=0.07	F1,33=0.12, p=0.73	F2,32=0.62, p=0.55
Hit RT ISI Change	F2,32=2.88, p=0.07	F1,33=2.16, p=0.15	F2,32=0.32, p=0.73
Hit SE ISI Change	F2,32=0.58, p=0.57	F1,33=0.28, p=0.60	F2,32=0.07, p=0.93
Digit span			
Forward	F2,32=1.15, p=0.33	F1,33=0.83, p=0.37	F2,32=0.06, p=0.94
Backward	F2,32=2.47, p=0.10	F1,33=0.09, p=0.77	F2,32=0.85, p=0.44
FTT			
Dominant finger	F2,32=3.84, p=0.032	F1,33=0.41, p=0.53	F2,32=0.59, p=0.56
Non-dominant finger	F2,32=0.02, p=0.98	F1,33=0.00, p=0.99	F2,32=0.04, p=0.96
TOL			
TOL accuracy	F2,32=1.95, p=0.16	F1,33=1.64, p=0.21	F2,32=0.84, p=0.44
TOL time	F2,32=2.56, p=0.09	F1,33=0.00, p=0.95	F2,32=1.05, p=0.36
TOL score	F2,32=5.83, p=0.007	F1,33=0.34, p=0.56	F2,32=0.56, p=0.58
CTT			
Color 1 (time in seconds)	F2,32=1.01, p=0.38	F1,33=0.06, p=0.80	F2,32=1.28, p=0.29
Color 2 (time in seconds)	F2,32=0.44, p=0.66	F1,33=0.91, p=0.35	F2,32=0.81, p=0.45
Stroop Interference Test			
Stroop Interference tendency			
Reading interference tendency	F2,32=0.13, p=0.88	F1,33=0.57, p=0.46	F2,32=0.11, p=0.90
Naming interference tendency	F2,32=0.25, p=0.78	F1,33=0.49, p=0.49	F2,32=0.05, p=0.96

The abbreviations and notes as in Table S2. Significant P-values for interaction effects are presented in bold.

Table S8. Cardiac autonomic measures over time

Autonomic parameters		Baseline		At the end of tACS		One-week follow-up		One-month follow-up	
		n	Mean (SD)	n	Mean (SD)	n	Mean (SD)	n	Mean (SD)
RR intervalrest	Active	18	782.50 (136.16)	17	853.00 (130.60)	17	821.00 (128.59)	17	811.35 (122.81)
	Sham	18	736.89 (129.91)	18	748.33 (126.01)	18	735.89 (126.99)	18	766.72 (129.99)
RR intervaltask-minus-rest	Active	18	-71.94 (32.90)	17	-25.53 (15.48)	17	-14.00 (26.87)	17	3.76 (26.91)
	Sham	18	-79.06 (30.31)	18	-59.33 (11.64)	18	-0.72 (29.19)	18	-10.66 (23.53)
HF-HRVrest	Active	18	3.99 (2.09)	17	4.76 (2.20)	17	4.46 (2.20)	17	4.43 (2.31)
	Sham	18	3.33 (1.66)	18	3.92 (1.68)	18	3.77 (1.65)	18	4.20 (1.51)
HF-HRVtask-minus-rest	Active	18	-0.32 (0.71)	17	-0.19 (0.11)	17	-0.40 (0.77)	17	-0.21 (0.54)
	Sham	18	-0.32 (0.38)	18	-0.46 (0.14)	18	-0.09 (0.69)	18	0.01 (0.55)

Abbreviations: tACS, online theta transcranial alternating current stimulation; SD, standard deviations; RR intervalrest, RR interval during resting conditions (ms); RR intervaltask-minus-rest, RR intervals during dual 2-back tasks minus RR intervals during resting conditions (ms); HF-HRVrest, high-frequency power of heart rate variability (HF-HRV) during resting conditions [ln(ms2)]; HF-HRVtask-minus-rest, HF-HRV during dual 2-back tasks minus HF-HRV during resting conditions [ln(ms2)].

Table S9. Baseline cardiac autonomic measures between active tACS and sham group

Variables	Active tACS (N=18) Mean ± SD	Sham (N=18) Mean ± SD	Statistics
RR intervalrest	782.50 ± 136.16	736.89 ± 129.91	Unadjusted: t=1.03, p=0.31; adjusteda: F=0.19, p=0.67
RR intervaltask-minus-rest	-71.94 ± 32.90	-79.06 ± 30.31	Unadjusted: t=0.67, p=0.51; adjusteda: F=0.21, p=0.65
HFrest	3.99 ± 2.09	3.33 ± 1.66	Unadjusted: t=1.06, p=0.30; adjusteda: F=0.57, p=0.46
HF-HRVtask-minus-rest	-0.32 ± 0.71	-0.32 ± 0.38	Unadjusted: t=-0.02, p=0.99; adjusteda: F=0.17, p=0.69

Abbreviations: tACS, online theta transcranial alternating current stimulation; SD, standard deviations; RR intervalrest, RR interval during resting conditions (ms); RR intervaltask-minus-rest, RR intervals during dual 2-back tasks minus RR intervals during resting conditions (ms); HF-HRVrest, high-frequency power of heart rate variability (HF-HRV) during resting conditions [ln(ms2)]; HF-HRVtask-minus-rest, HF-HRV during dual 2-back tasks minus HF-HRV during resting conditions [ln(ms2)].

aAdjusted for sex, age, body mass index, physical activity, smoking, hypertension, and diabetes mellitus.

Table S10. Associations between cardiac autonomic measures and improvement in negative symptoms in patients treated with active tACS

	Changes in PANSS_N scores (baseline to the end of tACS)		Changes in PANSS_N scores (baseline to one-week F/U)		Changes in PANSS_N scores (baseline to one-month F/U)	
Autonomic parameters	r	p	r	p	r	p
RR intervalrest at baseline	0.03	0.90	-0.33	0.18	-0.30	0.22
RR intervalrest changes from:						
baseline to the end of tACS	0.004	0.99	0.05	0.86	0.10	0.71
baseline to the one-week follow-up	NA	NA	0.11	0.68	0.21	0.42
baseline to the one-month follow-up	NA	NA	NA	NA	-0.14	0.61
RR intervaltask-minus-rest at baseline	0.76	<0.001	0.55	0.02	0.38	0.12

RR intervaltask-minus-rest changes from:						
baseline to the end of tACS	-0.79	<0.001	-0.60	0.012	-0.38	0.13
baseline to the one-week follow-up	NA	NA	-0.24	0.36	-0.22	0.40
baseline to the one-month follow-up	NA	NA	NA	NA	-0.45	0.07
HFrest at baseline	0.03	0.91	-0.26	0.30	-0.38	0.12
HFrest changes from:						
baseline to the end of tACS	-0.39	0.12	-0.07	0.79	0.08	0.75
baseline to the one-week follow-up	NA	NA	-0.07	0.79	0.08	0.75
baseline to the one-month follow-up	NA	NA	NA	NA	-0.05	0.86
HFtask-minus-rest at baseline	0.29	0.24	0.19	0.44	0.22	0.38
HFtask-minus-rest changes from:						
baseline to the end of tACS	-0.32	0.22	-0.22	0.39	-0.16	0.53
baseline to the one-week follow-up	NA	NA	-0.004	0.99	-0.13	0.62
baseline to the one-month follow-up	NA	NA	NA	NA	-0.11	0.68

Abbreviations: PANSS_N, negative symptoms subscale of the Positive and Negative Syndrome Scale; tACS, online theta transcranial alternating current stimulation; F/U, follow-up; NA, not assessed; Other abbreviations: see Table S8.

P values that reach the corrected significance level (false discovery rate method) are in bold.

3. Supplementary Figures

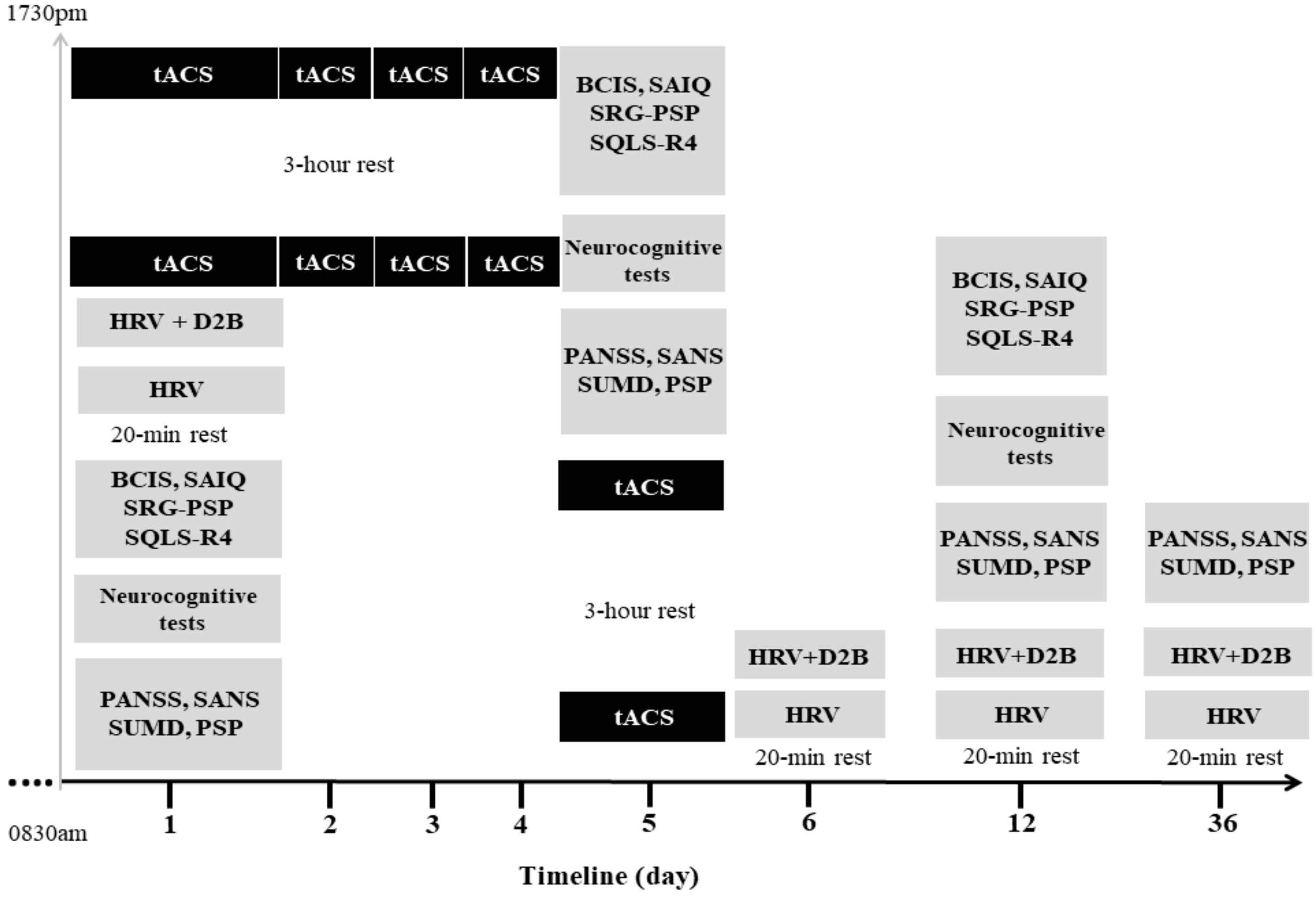

Figure S1. The flow chart of all the measurements conducted at baseline, after 10-session stimulation, and at the follow-up visits. PANSS, Positive and Negative Syndrome Scale; SANS, Scale for the Assessment of Negative Symptoms; SUMD, the abbreviated version of the Scale to Assess Unawareness in Mental Disorder in schizophrenia; PSP, the Taiwanese version of the Personal and Social Performance scale; BCIS, the Taiwanese version of the Beck's Cognitive Insight Scale; SAIQ, the Taiwanese version of the Self-Appraisal of Illness Questionnaire; SRG-PSP, Self-reported version of the graphic Personal and Social Performance scale; SQLS-R4, Schizophrenia Quality of Life Scale Revision Four; HRV, heart rate variability; tACS, online left-hemispheric in-phase frontoparietal theta transcranial alternating current stimulation during dual n-back training tasks.

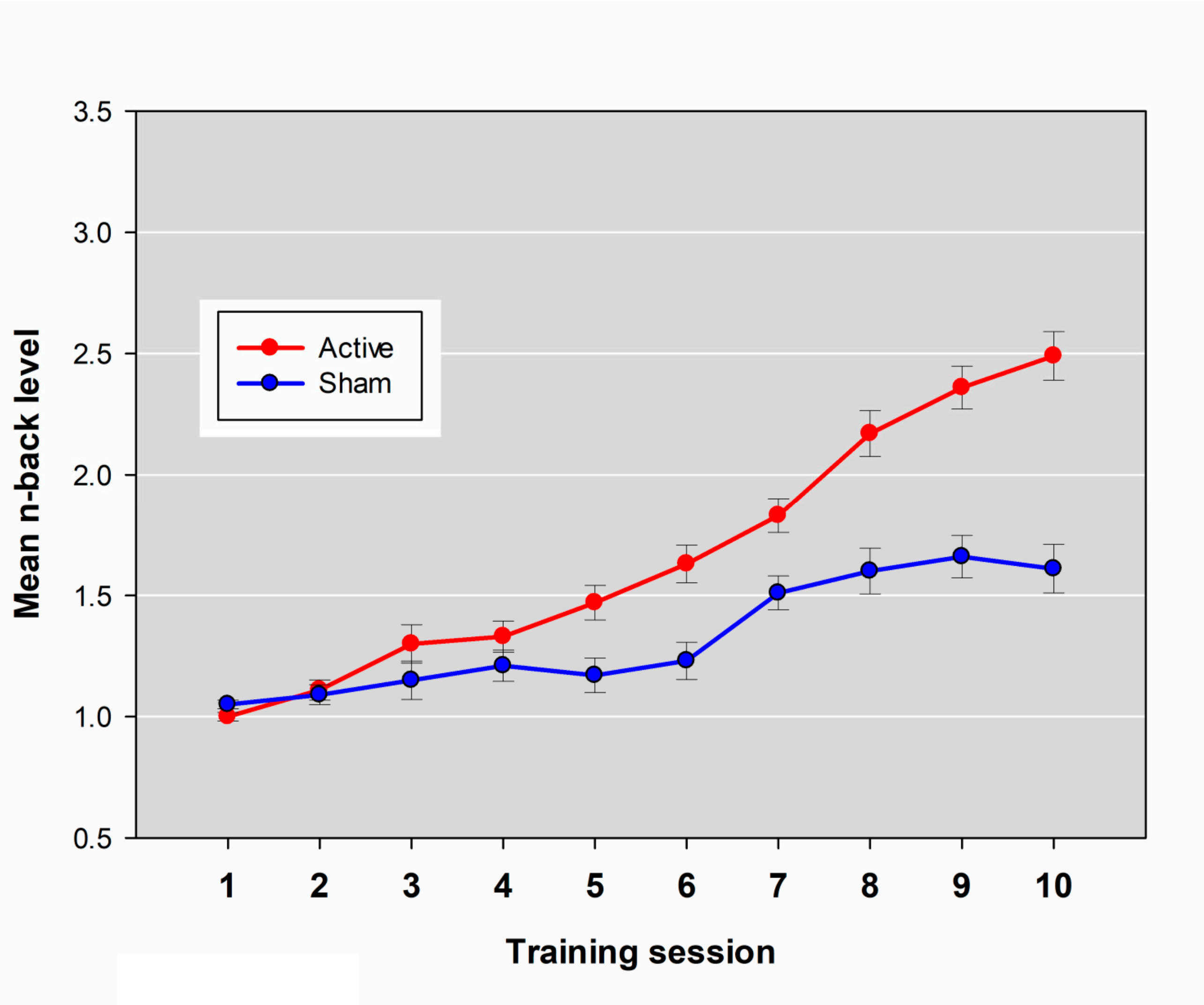

Figure S2. The mean level of n achieved on the dual n-back task across the 10 training sessions by the patients in the active tACS versus sham group was presented. The level of n is determined by the patients' performance. Analyses of the performance over time on each dual n-back training session showed a significant group-by-time interaction ($F_{9,26}=4.74$, $p=0.001$). The mean n-back level achieved by all the participants during the first training session was 1.03, which elevated to 2.05 in the final session. The active theta-tACS group showed more gains in n-back levels throughout the 10-session training compared to the sham group (1.49 versus 0.55, $p<0.001$). Error bars indicated the standard errors.

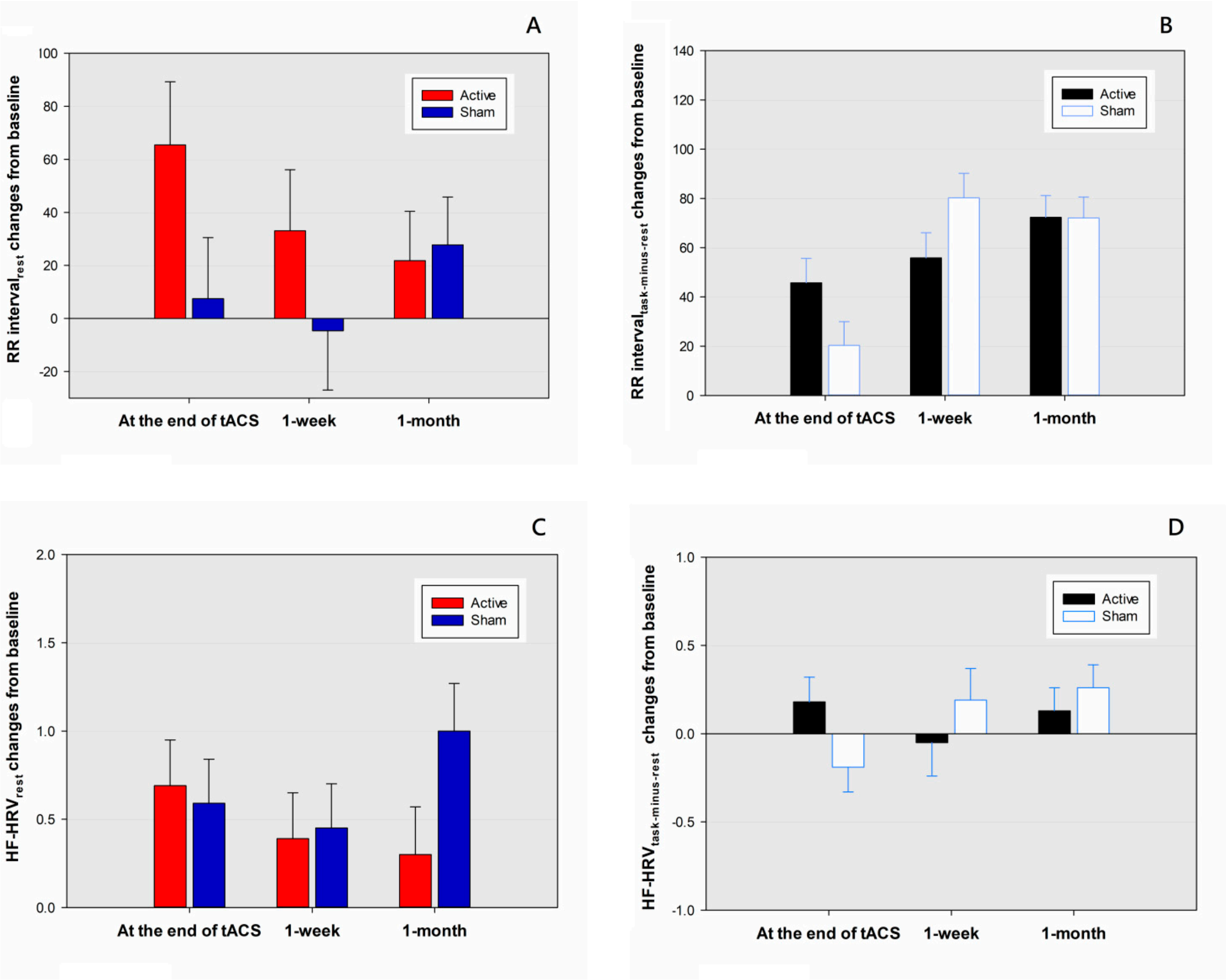

Figure S3. Changes from baseline in RR intervalrest (A), RR intervaltask-minus-rest (B), HF-HRVrest (C), and HF-HRVtask-minus-rest (D) between active stimulation group and sham group at postbaseline assessments of the end of theta-tACS, 1-week and 1-month follow-ups. Data were adjusted for covariates (sex, age, body mass index, physical activity, smoking, hypertension, and diabetes mellitus). Error bars indicated the standard errors. The significance of the between-group differences in all the indices did not survive false discovery rate (FDR) correction for multiple comparisons. RR intervalrest, RR interval during resting conditions (ms); RR intervaltask-minus-rest, RR intervals during dual 2-back tasks minus RR intervals during resting conditions (ms); HF-HRVrest, high-frequency power of heart rate variability (HF-HRV) during resting conditions [ln(ms2)]; HF-HRVtask-minus-rest, HF-HRV during dual 2-back tasks minus HF-HRV during resting conditions [ln(ms2)].

4. References

1. Palm, U., et al., Evaluation of sham transcranial direct current stimulation for randomized, placebo-controlled clinical trials. Brain Stimul 2013. 6, 690-5.
2. Jaeggi, S.M., et al., Improving fluid intelligence with training on working memory. Proc Natl Acad Sci U S A 2008. 105, 6829-33.
3. Kay, S.R., A. Fiszbein, and L.A. Opler, The positive and negative syndrome scale (PANSS) for

schizophrenia. Schizophr Bull 1987. 13, 261-76.
4. Wallwork, R.S., et al., Searching for a consensus five-factor model of the Positive and Negative Syndrome Scale for schizophrenia. Schizophr Res 2012. 137, 246-50.
5. van der Gaag, M., et al., The five-factor model of the Positive and Negative Syndrome Scale II: a ten-fold cross-validation of a revised model. Schizophr Res 2006. 85, 280-7.
6. Cella, M., et al., Effects of cognitive remediation on negative symptoms dimensions: exploring the role of working memory. Psychol Med 2017, 1-9.
7. Andreasen, N.C., The Scale for the Assessment of Negative Symptoms (SANS): conceptual and theoretical foundations. Br J Psychiatry Suppl 1989, 49-58.
8. Michel, P., et al., Psychometric properties of the abbreviated version of the Scale to Assess Unawareness in Mental Disorder in schizophrenia. BMC Psychiatry 2013. 13, 229.
9. Kao, Y.C. and Y.P. Liu, The clinical applicability of the Self-Appraisal of Illness Questionnaire (SAIQ) to chronic schizophrenic patients in Taiwan. Psychiatr Q 2010. 81, 215-25.
10. Marks, K.A., et al., Self-Appraisal of Illness Questionnaire (SAIQ): relationship to researcher-rated insight and neuropsychological function in schizophrenia. Schizophr Res 2000. 45, 203-11.
11. Kao, Y.C. and Y.P. Liu, The Beck Cognitive Insight Scale (BCIS): translation and validation of the Taiwanese version. BMC Psychiatry 2010. 10, 27.
12. Kao, Y.C., et al., Assessing cognitive insight in nonpsychiatric individuals and outpatients with schizophrenia in Taiwan: an investigation using the Beck Cognitive Insight Scale. BMC Psychiatry 2011. 11, 170.
13. Beck, A.T., et al., A new instrument for measuring insight: the Beck Cognitive Insight Scale. Schizophr Res 2004. 68, 319-29.
14. Wu, B.J., et al., Validation of the Taiwanese Mandarin version of the Personal and Social Performance scale in a sample of 655 stable schizophrenic patients. Schizophr Res 2013. 146, 34-9.
15. Morosini, P.L., et al., Development, reliability and acceptability of a new version of the DSM-IV Social and Occupational Functioning Assessment Scale (SOFAS) to assess routine social functioning. Acta Psychiatr Scand 2000. 101, 323-9.
16. Bai, Y.M., et al., The development of a self-reported scale for measuring functionality in patients with schizophrenia--self-reported version of the graphic Personal and Social Performance (SRG-PSP) scale. Schizophr Res 2014. 159, 546-51.
17. Kuo, P.J., et al., Validation of the Chinese version of the Schizophrenia Quality of Life Scale Revision 4 (SQLS-R4) in Taiwanese patients with schizophrenia. Qual Life Res 2007. 16, 1533-8.
18. Heaton, R.K., G.J. Chelune, and J.L. Talley, Manual, Wisconsin Card Sorting Test. 1993, Odessa, FL: Psychological Assessment Resources.
19. Lopez-Luengo, B., A. Gonzalez-Andrade, and M. Garcia-Cobo, Not All Differences between Patients with Schizophrenia and Healthy Subjects Are Pathological: Performance on the Conners' Continuous Performance Test. Arch Clin Neuropsychol 2016.
20. Wechsler, D., WAIS-III:Wechsler Adult Intelligence Scale, Administration and Scoring Manual,3rd Edn. 1997, San Antonio, Tex, USA: Psychological Corporation.

21. Lezak, M.D., Neuropsychological assessment. 3rd ed. 1995, New York, NY: Oxford University Press.
22. Garcia-Alba, J., et al., Validation and Normalization of the Tower of London-Drexel University Test 2nd Edition in an Adult Population with Intellectual Disability. Span J Psychol 2017. 20, E32.
23. Maj, M., et al., Evaluation of two new neuropsychological tests designed to minimize cultural bias in the assessment of HIV-1 seropositive persons: a WHO study. Arch Clin Neuropsychol 1993. 8, 123-35.
24. Scarpina, F. and S. Tagini, The Stroop Color and Word Test. Front Psychol 2017. 8, 557.
